# sourceR: Classification and source attribution of infectious agents among heterogeneous populations

**DOI:** 10.1371/journal.pcbi.1005564

**Published:** 2017-05-30

**Authors:** Poppy Miller, Jonathan Marshall, Nigel French, Chris Jewell

**Affiliations:** 1 CHICAS, Faculty of Health and Medicine, Lancaster University, Lancaster, England, United Kingdom; 2 Institute of Fundamental Sciences, Massey University, Palmerston North, New Zealand; 3 mEpiLab, Massey University, Palmerston North, New Zealand; 4 New Zealand Food Safety Science and Research Centre, Palmerston North, New Zealand; 5 New Zealand Institute for Advanced Studies, Auckland, New Zealand; Universite de Montreal, CANADA

## Abstract

Zoonotic diseases are a major cause of morbidity, and productivity losses in both human and animal populations. Identifying the source of food-borne zoonoses (e.g. an animal reservoir or food product) is crucial for the identification and prioritisation of food safety interventions. For many zoonotic diseases it is difficult to attribute human cases to sources of infection because there is little epidemiological information on the cases. However, microbial strain typing allows zoonotic pathogens to be categorised, and the relative frequencies of the strain types among the sources and in human cases allows inference on the likely source of each infection. We introduce sourceR, an R package for quantitative source attribution, aimed at food-borne diseases. It implements a Bayesian model using strain-typed surveillance data from both human cases and source samples, capable of identifying important sources of infection. The model measures the force of infection from each source, allowing for varying survivability, pathogenicity and virulence of pathogen strains, and varying abilities of the sources to act as vehicles of infection. A Bayesian non-parametric (Dirichlet process) approach is used to cluster pathogen strain types by epidemiological behaviour, avoiding model overfitting and allowing detection of strain types associated with potentially high “virulence”. sourceR is demonstrated using *Campylobacter jejuni* isolate data collected in New Zealand between 2005 and 2008. Chicken from a particular poultry supplier was identified as the major source of campylobacteriosis, which is qualitatively similar to results of previous studies using the same dataset. Additionally, the software identifies a cluster of 9 multilocus sequence types with abnormally high ‘virulence’ in humans. sourceR enables straightforward attribution of cases of zoonotic infection to putative sources of infection. As sourceR develops, we intend it to become an important and flexible resource for food-borne disease attribution studies.

This is a *PLOS Computational Biology* software paper.

## Introduction

Zoonotic diseases are a major source of human morbidity world wide. In 2010, there were an estimated 600 million cases globally [1], of which 96 million were *Campylobacter spp*. resulting in 21,000 deaths [2]. Attributing cases of food-borne disease to putative sources of infection is crucial for identifying and prioritising food safety interventions, prompting routine national recording of human cases and surveillance of high-risk sources in many countries—for example FoodNet in the US [3], the Danish Zoonosis Centre (food.dtu.dk), and the Ministry for Primary Industries in New Zealand (foodsafety.govt.nz).

Traditional approaches to source attribution include observational risk assessment, extrapolation of surveillance or outbreak data, and epidemiological field studies [4]. The results of such direct observational methods may be highly uncertain due to long and variable disease incubation times, and many exposures of an individual to multiple sources of infection. Nevertheless, statistical modelling of human case count data, incorporating molecular strain typing of pathogen isolates from national surveillance programmes, has shown promise for identifying important sources of food-borne illness [5, 6].

The aim of this paper is to extend current approaches to statistical source attribution, and to provide a standard software package, sourceR, providing an intuitive interface to source attribution models for epidemiological domain specialists. Our principle innovation is a novel class of Bayesian non-parametric source attribution model which classifies strain types by differential epidemiological behaviour and accurately quantifies uncertainty. Furthermore, we allow for spatial and temporal heterogeneity in case and source data with the aim of detecting differential exposures to infection sources across space and time. sourceR represents the first standard software for source attribution, and is designed for use by epidemiologists and public health decision makers. It is written as an add-on package to R, the open-source lingua-franca for modern epidemiological analysis, and incorporates an object-orientated style to facilitate further model development and future maintainability.

The paper is structured as follows. We first introduce a motivating example and review existing source attribution models. The new model is described in the Design and Implementation section followed by a demonstration of model fitting using sourceR in the Materials and Methods section. Results and Discussion sections follow, and it concludes with details of Availability and Future directions.

### Example: *Campylobacter* food-poisoning in Manawatu, New Zealand

In 2006, New Zealand had one of the highest incidences of campylobacteriosis in the developed world, with an annual incidence in excess of 400 cases per 100,000 people [7]. Our motivating data set was collected between 2005 and 2008 in the Manawatu region of New Zealand with the aim of identifying the most important sources of campylobacteriosis and implementing interventions. A campaign to change poultry processing procedures, supported in part by results from previous quantitative source attribution approaches, was successful in leading to a sharp decline in campylobacteriosis incidence after 2007 [6].

*Campylobacter* has many subtypes which are usually defined using Multilocus Sequence Typing (MLST), a commonly used genotyping method providing a relatively rapid method of characterising isolates. An MLST sequence type is a unique combination of alleles at specified gene loci, typically located in conserved regions of the genome [8, 9]. The data set consists of the dominant MLST-genotype *Campylobacter* isolated from each source (potential food and environmental sources) and human sample. The data was first published in [10], and is described in detail (including data collection methods) in [11] and [12]. These data are included in our sourceR package (named campy). We use this data set as a case study, and compare our results with previously published statistical approaches.

### Existing methods of source attribution

The general structure of the source attribution model is that the observed case-counts *y*_*i*_ for strain *i* (occurring in a defined surveillance period) are mutually independent Poisson distributed with means

$$\begin{array}{c} \lambda_{i}=\sum_{j=1}^{m}\alpha_{j}p_{ij}. \end{array}$$ (1)

where *p*_*ij*_ is the prevalence of strain *i* in source *j*, and “source effects” ***α*** measure each source’s capacity to act as a vehicle of infection. The estimated number of cases attributed to a particular source *j* is

$$\begin{array}{c} {\hat{\xi }}_{j}={\hat{\alpha }}_{j}\sum_{i=1}^{n}p_{ij}. \end{array}$$ (2)

Comparing the relative magnitudes of ${\hat{\xi }}_{j}$ provides a statistical method to prioritise intervention strategies to the most important sources of infection. The model is fitted in a Bayesian framework as posteriors for functions of parameters (such as *ξ*) are easily calculated, and to allow previous knowledge to be incorporated via informative priors.

A significant problem is that this model does not allow for some strain types have differential affinities for human infection resulting in over-dispersion of ***y***. Additionally, it does not allow for uncertainty in ***P***, inherent in sample based source data. In the rest of this section, we review current extensions to Eq 1 aimed at accounting for the Poisson over-dispersion in observed case numbers, and incorporating uncertainty in source surveillance data. In particular, the preliminary developments made by Hald *et al*. [5] and Müllner *et al*. [6] form an ontology on which we base our innovations.

#### Over-dispersion

Hald *et al*. [5] address the issue of Poisson over-dispersion in Eq 1 by introducing a “type effect” ***q*** accounting for some strain types being more adapted to human infection than others.

$$\begin{array}{c} \lambda_{i}=q_{i}\sum_{j=1}^{m}\alpha_{j}c_{j}p_{ij}. \end{array}$$ (3)

Additionally, they include an offset ***c*** representing known rates of consumption of each source foodstuff, allowing ***α*** to be interpreted as a source-specific factor independent of exposure. However, the addition of ***q*** as a vector of uncorrelated unknowns over-specifies the model, with *m* + *n* parameters but only *n* independent disease case count observations. Hald *et al*. therefore reduce the number of parameters by heuristic *a priori* grouping of the elements of ***q***, albeit with the generally undesirable property that quantification of uncertainty in the most appropriate choice of grouping is not readily permissible.

The “Modified Hald” model of Müllner *et al*. [6] treats ***q*** as log Normally distributed random effect, with unit mean and unknown variance *τ*^2^

$$\begin{array}{c} q_{i}∼\text{logNormal}\left(1,\tau^{2}\right) \end{array}$$ (4)

with a Gamma-distributed prior distribution imposed on *τ*^2^. However, this approach suffers from *a posteriori* non-identifiability of ***q*** and *τ*^2^, hindering the performance of MCMC algorithms used to fit the model [13]. Though this may be ameliorated by choosing an informative prior for *τ*^2^ with small mean, it results in severe shrinkage of ***q*** and inference which is sensitive to the choice of prior.

#### Uncertainty in source sampling

The Modified Hald model introduces uncertainty into the prevalences *p*_*ij*_ by modelling the source sampling process. Let *s*_*j*_ denote the total number of source samples collected from source *j* = 1, …, *m*, of which *x*_*ij*_ are positive for pathogen type *i*. Normalisation of the number of positive samples *x*_*ij*_ gives the relative prevalence $r_{ij}=x_{ij}/\sum_{i=1}^{n}x_{ij}$ of type *i* in source *j*. The relative prevalence *r*_*ij*_ is then combined with the prevalence of positive samples $k_{j}=\sum_{i=1}^{n}x_{ij}/s_{j}$ to calculate the absolute prevalence *p*_*ij*_ = *r*_*ij*_ × *k*_*j*_ of strain *i* in source *j*. The Modified Hald model was fitted in WinBUGS using an approximate two stage process [6]. First, a posterior distribution was estimated for the absolute prevalence of source types ***p***, using the model specified in Eqs (5) and (6):

$$\begin{array}{c} r_{\cdot j}∼\text{Dirichlet}\left(1\right)\;\forall \;j \end{array}$$ (5)

$$\begin{array}{c} k_{j}∼\text{Beta}\left(1,1\right)\;\forall \;j \end{array}$$ (6)

The marginal posterior for each element of ***p*** was then approximated by a Beta distribution

$$\begin{array}{c} p_{ij}∼\text{Beta}\left(w_{ij},v_{ij}\right) \end{array}$$

(using the method of moments to calculate *w*_*ij*_ and *v*_*ij*_) which was then used as an independent prior in step 2. Since each isolate is assigned to only one type, we must observe $\sum_{i=1}^{n}r_{ij}=1$, and therefore $\sum_{i=1}^{n}p_{ij}=k_{j}$. This is not enforced when using independent Beta priors for each *p*_*ij*_ which results in *k*_*j*_ (the probability of a sample being positive given the sample is from source *j*) no longer being constrained to be between 0 and 1.

## Design and implementation

Our approach addresses the deficiencies inherent in both the Hald and Modified Hald models by fitting a joint model for both source and human case sampling with non-parametric clustering of the type effects. This allows integration over uncertainty in the source sampling process without resorting to an approximate marginal probability distribution on ***p***. The overdispersion is solved by non-parametrically clustering the pathogen types using a Dirichlet process (DP) on the type effect vector ***q***. This is a data driven, automatic method which reduces the effective number of parameters in the model without requiring strong assumptions about *τ*^2^ in Eq 4. Additionally, it quantifies the similarity between epidemiological characteristics (virulence, pathogenicity and survivability) of the subtypes forming the basis of future research on the genetic determinants of this behaviour. Often, human case data is associated with location such as urban/rural, or GPS coordinates whilst food samples are likely to be less spatially constrained (due to distances between production and sale locations). Both human and source data may exist for multiple time-periods. Therefore, we allow for spatial and temporal heterogeneity in the data.

### HaldDP model

As with the Hald model, we assume the number of human cases *y*_*itl*_ identified by isolation of subtype *i* in time-period *t* at location *l* is Poisson distributed

$$\begin{array}{c} y_{itl}∼\text{Poisson}\left(\lambda_{itl}=q_{i}\sum_{j=1}^{m}\alpha_{jtl}p_{ijt}\right) \end{array}$$ (7)

We allow for different exposures of humans to sources in different locations and times, by allowing the source effects to vary between times and locations, *α*_*jtl*_.

For each source *j*, we model the number of positive source samples

$$\begin{array}{c} x_{jt}∼\text{Multinomial}\left(s_{jt}^{+},r_{jt}\right) \end{array}$$ (8)

where ***x***_*jt*_ = (*x*_*ijt*_, *i* = 1,…,*n*)^*T*^ denotes the vector of type-counts in source *j* in time-period *t*, $s_{jt}^{+}=\sum_{i=1}^{n}x_{ijt}$ denotes the number of positive samples obtained, and ***r***_*jt*_ denotes a vector of relative prevalences *Pr*(type_*i*_|source_*j*_, time_*t*_). This automatically places the constraint $\sum_{i=1}^{n}r_{ijt}=1$. The source case model is then coupled to the human case model through the simple relationship

$$\begin{array}{c} p_{ijt}=r_{ijt}k_{jt} \end{array}$$ (9)

where *k*_*jt*_ is the prevalence of any isolate in source *j* in time-period *t*.

In principle, a Beta distribution could be used to model *k*_*jt*_, arising as the conjugate posterior distribution of a Binomial sampling model for $s_{jt}^{+}$ positive samples from *s*_*jt*_ tested, and a Beta prior on *k*_*jt*_. We instead choose to fix the source prevalences at their empirical estimates ($k_{jt}=s_{jt}^{+}/s_{jt}$) because the number of source samples is typically high.

The type effects ***q***, which are assumed invariant across time or location, are drawn from a DP with base distribution *Q*_0_ and a concentration parameter *a*_*q*_

$$\begin{array}{c} q_{i}∼\text{DP}\left(a_{q},Q_{0}\right). \end{array}$$ (10)

The Dirichlet process is a probability distribution whose range is a set of probability distributions and is defined by a base distribution and concentration parameter [14]. The concentration parameter of the DP *a*_*q*_ encodes prior information on the number of groups *K* to which the pathogen types are assigned. The Gamma base distribution of the DP *Q*_0_ induces a prior for the cluster locations. The DP groups the elements of ***q*** into a finite set of clusters 1: *κ* (unknown *a priori*) with values *θ*_1_,…,*θ*_*κ*_ which addresses the inevitable over-dispersion in the case counts ***y*** robustly and clusters subtypes into groups with similar epidemiological behaviour.

Heterogeneity in the source matrix *x* is required to identify clusters from sources, which may not be guaranteed *a priori* due to the observational nature of the data collection.

### Inference

This section describes how the model is fitted in a Bayesian context by first describing the McMC algorithm used to fit this model, then developing the prior model.

#### McMC algorithm

The joint model over all unobserved and observed quantities is fitted using Markov chain Monte Carlo (McMC, full details in S1 Appendix). The source effects and relative prevalence parameters are updated using independent adaptive Metropolis-Hastings updates [15]. The type effects ***q*** are modelled using a DP (Eq 10) with a Gamma base distribution *Q*_0_ ∼ *Gamma*(*a*_*θ*_,*b*_*θ*_). The choice of a Gamma base distribution with the Poisson likelihood (Eq 7) permits the use of a marginal Gibbs strategy for efficient sampling from the posterior ditribution of ***q***. Each observation *i* is assigned to a cluster *k* with value *θ*_*k*_, such that *q*_*i*_ ↦ *θ*_*k*_. The algorithm proceeds by alternately sampling from the posterior of the group assignments (adding new clusters or deleting empty clusters as necessary), and the posterior of *θ*_*k*_ for each cluster.

#### Priors

The parameters ***α***_*tl*_ and ***q*** account for a multitude of source and type specific factors which are difficult to quantify *a priori*. Therefore, with no single real-world interpretation, the distributional form of the priors were chosen for their flexibility. A Dirichlet prior is placed on each **r**_*jt*_ which suitably constrains the individuals *r*_*ij*_s such that $\sum_{i=1}^{n}r_{ijt}=1$. A Dirichlet prior is also placed on each *α*_*tl*_, with the constraint $\sum_{j=1}^{m}\alpha_{jtl}=1$ aiding identifiability between the mean of the source and type effect parameters. In sourceR, the concentration parameter of the DP *α*_*q*_ is specified by the analyst as a modelling decision.

We note that the choice of base distribution *Q*_0_ may have a stronger effect than anticipated due to the small size of the relative prevalence and source effect parameters. This can been seen by considering the marginal posterior for *θ*_*k*_

$$\begin{array}{c} \theta_{k}∼\text{Gamma}\left(a_{\theta }+\sum_{i:S_{i}=k}y_{i},b_{\theta }+\sum_{i:S_{i}=k}\sum_{j=1}^{m}\alpha_{j}\cdot p_{ij}\right) \end{array}$$

The term $\sum_{i:S_{i}=k}\sum_{j=1}^{m}\alpha_{j}\cdot p_{ij}$ is very small (due to the Dirichlet priors on *α* and **r**_*j*_), which can result in even a fairly small rate parameter (*b*_*θ*_) dominating.

### Code implementation

Standard McMC packages (e.g. WinBUGS, Stan, PyMC3) cannot implement marginal Gibbs sampling for Dirichlet processes, necessitating a custom McMC framework (see section ‘Extensibility’). We chose R as a platform because of its ubiquity in epidemiology, and advanced support for post-processing of McMC samples. Dependencies on other R packages are required, but these are installed automatically by R’s package manager.

sourceR uses an object-oriented design, which allows separation of the model from the McMC algorithm. Internally, the model is represented as a directed acyclic graph (DAG) in which nodes are represented by an R6 class hierarchy. Generic adaptive Metropolis Hastings algorithms are attached to each parameter node, with the conditional independence properties of the DAG allowing automatic computation of the required (log) conditional posterior densities.

A difficulty with the DAG setup is the representation of the DP model on the type effects ***q***, since each update of the marginal Gibbs sampler requires structural alterations. Therefore, we subsume the entire DP into a single node, with a bespoke marginal Gibbs sampling algorithm written for our Gamma base-distribution and Poisson likelihood model.

## Materials and methods

The case study below illustrates how the sourceR package is used in practice. We compare the results of our approach with results from the Modified Hald, Asymmetric Island (see S2 Appendix and [16, 17]), and the “Dutch” model (see S3 Appendix and [18]). The priors for our model were selected to be minimally informative. The prevalence *k*_*j*_ is calculated by dividing the number of positive samples by the total number of samples for each source. In the data below, we note that for several samples the MLST typing failed, with the number of positive samples exceeding the apparent total number of MLST-typed isolates. Assuming MLST typing fails independently of pathogen type, this does not bias our results.

The model fitting process begins by formatting the data, constructing the HaldDP model and setting the McMC parameters before running the algorithm using the update() method.

*## Format data*

y <- Y(data = campy$cases, *# Cases*

 y = “Human”, type = “Type”, time = “Time”, location = “Location”)

x <- X(data = campy$sources, *# Sources*

 x = “Count”, type = “Type”, time = “Time”, source = “Source”)

k <- Prev(data = campy$prev, *# Prevalences*

 prev = “Value”, time = “Time”, source = “Source”)

*## Set priors*

priors = list(a_theta = 0.01, b_theta = 0.00001, a_alpha = 1, a_r = 0.1)

*## Construct model*

my_model <- HaldDP(y = y, x = x, k = k, priors = priors, a_q = 0.1)

*## Set mcmc parameters*

my_model$mcmc_params(n_iter = 1000, burn_in = 10000, thin = 500)

*## Run model*

my_model$update()

The sourceR package provides methods to extract and subset the complex posterior, calculate medians and credible intervals (with three possible methods percentile, SPIn [19], or Chen-Shao [20]) and plot a heatmap with a dendrogram showing the clustering of the type effects.

my_model$extract()

my_model$summary(alpha = 0.05, CI_type = “percentiles”)

my_model$plot_heatmap()

## Results

**Fig 1:**
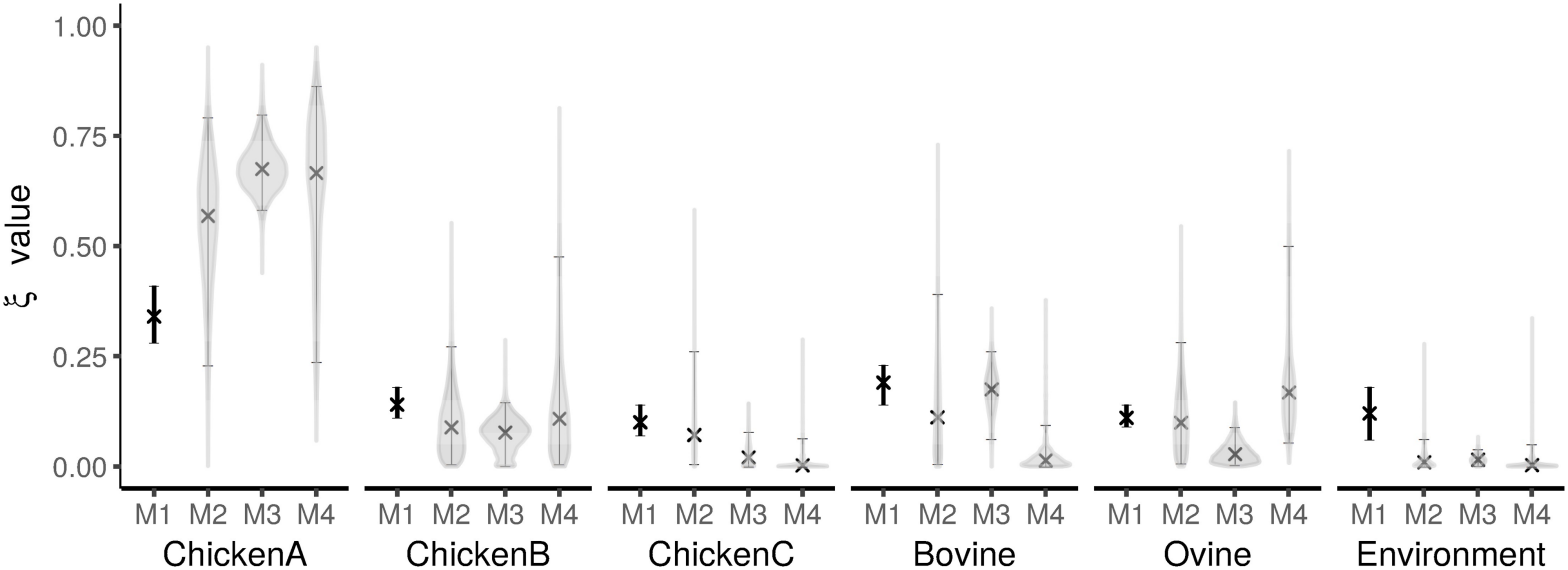
Comparison of the proportion of human campylobacteriosis cases attributable to each source. The models compared are: M1 (Dutch model), M2 (Modified Hald model), M3 (Island model) and M4 (HaldDP model). Error bars represent 95% percentile confidence or credible intervals with medians shown as a cross. Violin plots show the marginal posteriors of the *ξ*_*j*_ parameters.

Fig 1 shows the the proportion of cases attributed to each source. The HaldDP model identified the highest proportion of human campylobacteriosis cases as coming from chicken produced by supplier A (a median of 67 percent of cases attributed). A further 11 percent were attributed to Chicken from poultry supplier B and 17 percent to Ovine. The median values for the proportion of cases attributed to each source are qualitatively similar between all models except the Dutch method.

**Fig 2:**
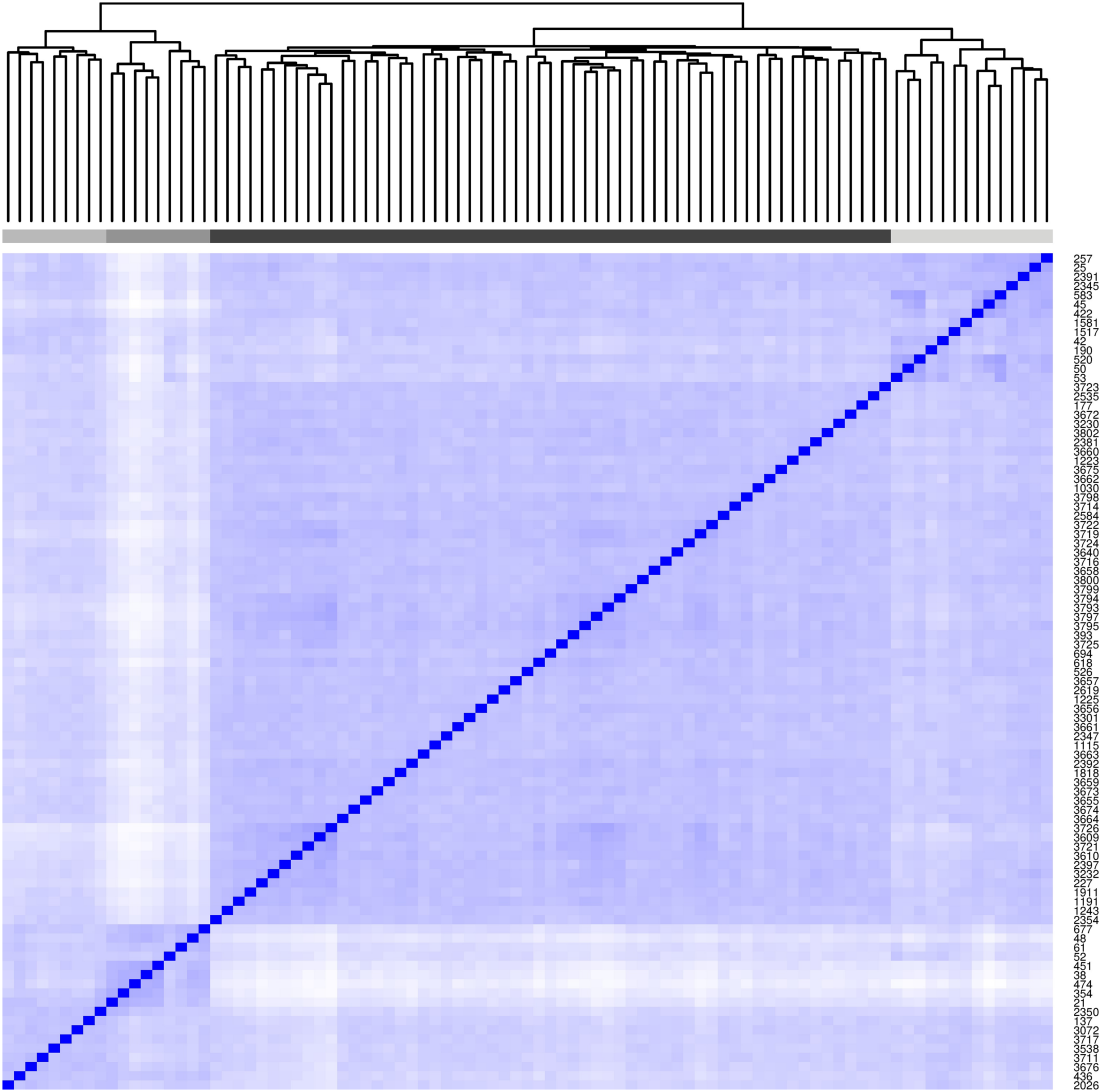
Heatmap showing the grouping of the type effects (q). A white pixel represents a dissimilarity value of 1 between a pair of sub types, whilst dark blue (see pixels on the diagonal) gives a value of zero. The grey coloured bar shows the groupings if the dendrogram is cut at 4 groups.

**Fig 3:**
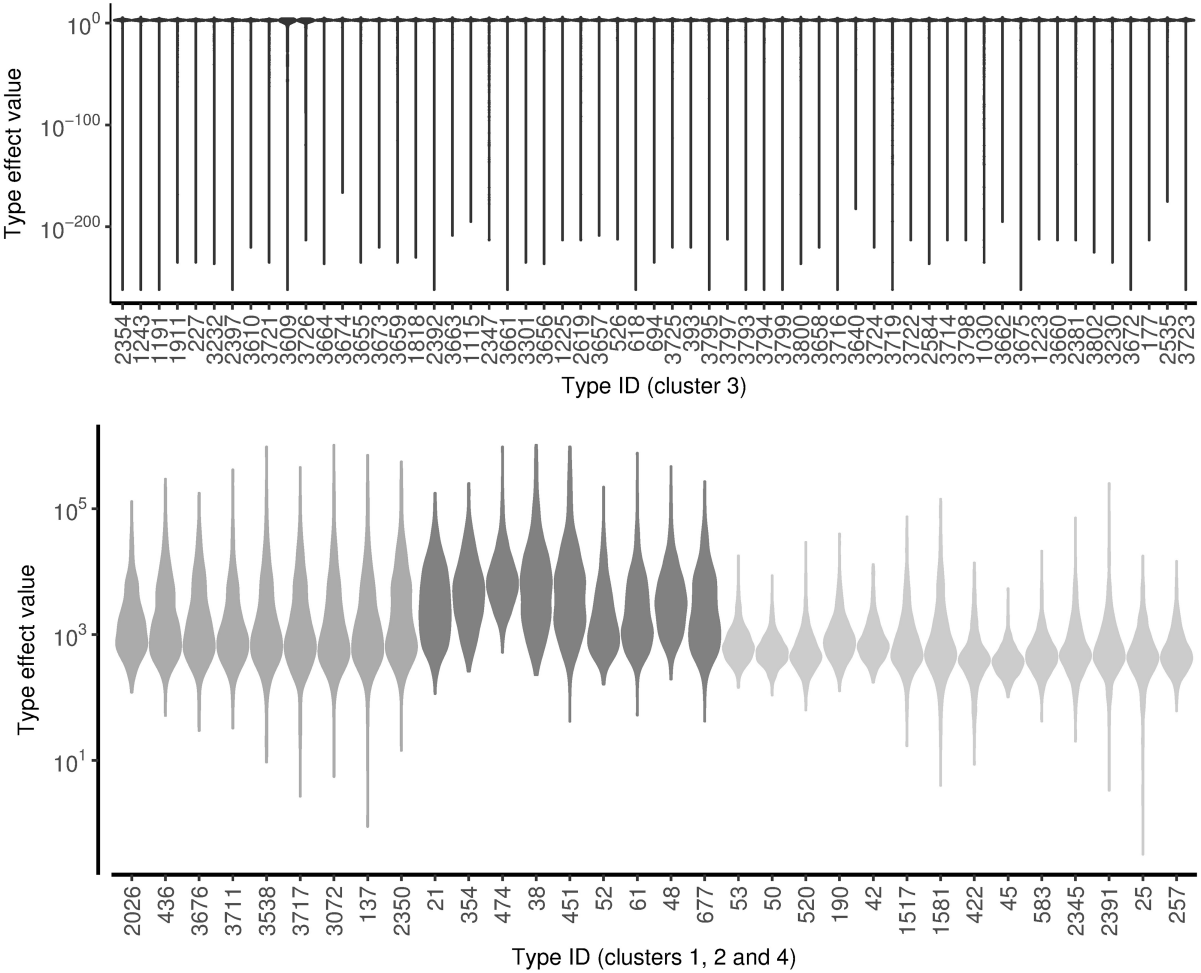
Violin plots of the marginal distributions of the type effects (q). Note that the y axis uses a a log scale axis. The fill colour matches the coloured grouping bar on the heatmap.

To visualise how the DP has clustered the type effects, Gower’s distance [21] is used to compute a dissimilarity matrix between all pairs of types. Fig 2 shows that the DP identified four main type clusters (from 91 types). The violin plots of the marginal posterior distributions for each type effect (Fig 3) show the largest group of types has very small type effects and wide credible intervals compared to the other groups.

Model fit and convergence was assessed visually using trace and autocorrelation plots (see Fig A and Fig B in S4 Appendix).

## Discussion

sourceR represents a significant advance in source attribution modelling, and translation of advanced statistical methods into mainstream epidemiological use. In particular, the DP clustering results in a large decrease in the effective number of parameters in the model and allows detection of unusually virulent subtypes (group 2 in Fig 3) by epidemiological behaviour. The subtypes in each cluster have similar epidemiological traits (such as virulence, pathogenicity and survivability) which forms the basis for future research on genetic determinants of those traits. Additionally, if a particular type moved into the high virulence group when repeating the analysis with further data from a later time period, it would flag that type as possibly evolving to become more risky for humans. The type effects for group 3 subtypes have very wide credible intervals due to the sparsity of source samples and human cases for those types.

The relatively large uncertainty for the disease origin (the credible intervals of ***ξ***) is likely due to *C. jejuni’s* complex epidemiology [6] giving rise to *a posteriori* correlations between components of ***α*** and ***q***. This is expected due to bias/variance trade-off: the Dutch and Island models both lack type effects risking biased results due to not all types being equally likely to infect humans. The Island model also possesses inherently strong and difficult to verify *a priori* assumptions (see [16] and S2 Appendix) which are not subject to uncertainty quantification. Moreover, by removing the approximation inherent in the Modified Hald model, we expect the HaldDP model to more accurately reflect inferential uncertainty—this is particularly important for decision making in food hygiene policy, especially when commercial interests must be supported by rigorous scientific advice.

Mixing and *a posteriori* correlations of the HaldDP model are significantly decreased in comparison to the Modified Hald model, if not entirely resolved. Although heterogeneity in *X* is required to fit the models, a sparse or highly unbalanced source matrix increases posterior correlations between some source and type effects. In our experience, the algorithm works best when the source matrix has a moderate amount of heterogeneity.

Whilst the HaldDP results for ***ξ*** are qualitatively similar to those from the other models (Fig 1, we note an interesting disagreement between the Island and Hald model derivatives when comparing the the number of cases attributed to Ovine and Bovine. We conjecture that this may be due to some non-identifiability between Bovine and Ovine sources as both sources have high contamination from the same types increasing the sensitivity of ***ξ*** to sampling error. It may also be due to lack of explicit source and type effects in the Island model. Resolving this disparity is the subject of ongoing research.

## Availability and future directions

The stable release version of sourceR is available from the Comprehensive R Archive Network, released under a GPL-3 licence. The development version is available at http://fhm-chicas-code.lancs.ac.uk/millerp/sourceR. As this package develops, we intend sourceR to become a platform for new source attribution model development, providing a central analytic resource for public health professionals.

The main focus of extending sourceR will be on modelling spatiotemporal correlation in the time and location dependent parameters. A spatiotemporal correlation model on ***α***_*tl*_ could be used to identify particular foci of source contamination, enabling targeted investigation of particular food supply regions. Implementation of time varying type effects may be appropriate as *Campylobacter* can evolve quickly and genetic variation conferring virulence may not be apparent from coarse-scale MLST typing [22]. Interaction terms between some sources and types would allow for the biologically plausible possibility that certain types are differentially likely to survive and cause disease, dependent on the food source they appear in. Additionally, water/ environmental samples could be attributed to the other sources of infection allowing estimation of the proportion of cases attributed to different paths of infection (direct infection from the source versus infection via the environment).

However, including interaction terms and additional paths of infection would significantly increase the number of parameters and the number and strength of posterior correlations. With higher posterior correlations, the current Metropolis-Hastings based fitting algorithm would suffer from a loss of efficiency. This could be addressed with gradient-based fitting algorithms such as Hamiltonian Monte Carlo (HMC) [23] which are designed to converge to high-dimensional, non-orthogonal target distributions much more quickly. In particular, the No U-Turn Sample (NUTS) presents an attractive method for tuning HMC adaptively, a quality which we consider necessary to minimise user intervention and maximise research productivity [24].

With increased interest in source attribution models for both food-borne pathogens, sourceR has been written with extensibility in mind. In particular, the DAG representation allows for rapid construction of modified and new models. The package routines are written in R (as opposed to C or C++) to aid readability, with the node class hierarchy and three stage workflow designed to aid the addition of new model classes. All internal classes and methods are documented to enable prospective developers to familiarise themselves with the source code quickly, and an extensive test suite is provided. We note that the DAG framework is not limited solely to source attribution models and may used for other Bayesian applications, particularly those for which a Dirichlet process is required.

## Conclusions

We have presented a novel source attribution model which builds upon, and unites, the Hald and Modified Hald approaches. It is widely applicable, fully joint, and does not require approximations or a large number of assumptions. Mixing and *a posteriori* correlations are significantly decreased in comparison to the Modified Hald model. Furthermore, it allows the data to inform type effect clustering using a Bayesian non-parametric model which identifies groups of sub types with similar putative virulence, pathogenicity and survivability. This is a significant improvement over the previous attempts to improve model identifiability (fixing some source and type effects *a priori*, or modelling the type effects as random using a 2 stage model). Like the Modified Hald model, the new model incorporates uncertainty in the prevalence matrix into the model, however, it does this by fitting a fully joint model rather than a 2 step model. This has the advantage of allowing the human cases to influence the uncertainty in the source data and preserves the restriction on the sum of the prevalences for each source. The sourceR package implements this model to enable straightforward attribution of cases of zoonotic infection to putative sources of infection by epidemiologists and public health decision makers.

## Supporting information

S1 Appendix Full McMC algorithm. (PDF)

S2 Appendix Island model overview. (PDF)

S3 Appendix Dutch model overview. (PDF)

S4 Appendix Model fit and convergence diagnostic plots. (PDF)
