# Supplementary material for: sourceR: Classification and source attribution of infectious agents among heterogeneous populations
Source: PLoS Comput Biol. 2017 May 30;13(5):e1005564. doi: 10.1371/journal.pcbi.1005564 (PMC5473572; doi:10.1371/journal.pcbi.1005564)
Supplement: S1 Appendix — (PDF) [file pcbi.1005564.s001.pdf]

S1 Appendix. Full McMC Algorithm. This section gives the full details of the algorithm used to fit our fully joint non-parametric source attribution model. The outline McMC is shown in Algorithm 1.

Data: Human cases $\boldsymbol{y}$, source isolates $\boldsymbol{X}$, source prevalence $\boldsymbol{s}$

Initialize all parameters ;
for *z times* **do**
    foreach t, l **do**
1   Update $\boldsymbol{\alpha}_{tl}$;
    end
    foreach j,t **do**
2   Update $\boldsymbol{r}_{jt}$;
    end
3   Update $\boldsymbol{q}$;
    Save chain state ;
end

Algorithm 1: Outline McMC algorithm for the `HaldDP` model.

The Dirichlet distributed source effects $\boldsymbol{\alpha}_{tl}$ across times t and locations l (Step 1), and the relative prevalences $\boldsymbol{r}_{jt}$ across sources j and times t (Step 2) are updated using a constrained adaptive multisite logarithmic Metropolis-Hastings update step for 95% of proposals, and a constrained adaptive multisite Metropolis-Hastings update step for the remainder to prevent the chain getting stuck at very low values [1]. The adaptive algorithm updates the tuning value every 50 updates of the parameter. This is further explained in Algorithm 2.

For the Dirichlet process prior on $\boldsymbol{q}$, a marginal Gibbs sampler is constructed, as described in Algorithm 3. Let $\mathcal{H}$ denote a set of cluster identifiers, with the n-dimensional group assignment vector $\boldsymbol{c}$ associating elements of $\boldsymbol{q}$ with clusters, such that $c_i = h$ assigns q_i to cluster h. Furthermore, each cluster h assumes a value θ_h such that $q_i = \theta_{c_i}$.

In Step 1 of Algorithm 3, conjugacy between the Gamma-distributed base distribution P_0 and the Poisson data likelihood permits the calculation of Multinomial conditional posteriors for elements of $\boldsymbol{c}$ arising from the Chinese Restaurant Process construction. Here, the conditional posterior probability of type i being assigned to group h is as shown in Algorithm 3, with conjugacy permitting marginalisation with respect to the base distribution in order to calculate the probability of being assigned to a new group $h^\star$

$$p_{h^\star} = a_q \int_\Theta L(y_i|\theta, \lambda_i^\star) dP_0(\theta) = \frac{b_\theta^{a_\theta}(a_\theta + y_i)}{\Gamma(a_\theta)(b_\theta + \lambda_i^\star)^{a_\theta + y_i}}$$

with $y_i^\star = \sum_{t,l} y_{itl}$ and $\lambda_i^\star = \sum_{t,l} \boldsymbol{\alpha}_{tl}^T (\boldsymbol{r}_{it} \odot k_t)$

If a type is assigned to a new group, the set $\mathcal{H}$ is augmented and a corresponding cluster value is drawn from the posterior of $\theta_{h^\star}$. Conversely, $\mathcal{H}$ is shrunk if a particular group becomes empty.

In Step 2, the group values are drawn from the posterior, conditional on $\boldsymbol{c}$. The algorithm therefore alternates between updating group assignments $\boldsymbol{c}$ and group values $\boldsymbol{\theta}$. Hence, it explores the number of groups present, the type effects assigned to each group, and the values of each group.

Input: d-dimensional Dirichlet($\boldsymbol{a}$) distributed random variable $\boldsymbol{W}$, tuning variance vector $\boldsymbol{\sigma}$, online acceptance rate vector $\boldsymbol{\rho}$, z the current McMC iteration number.
Output: Updated $\boldsymbol{W}$ and $\boldsymbol{\sigma}$.

Let $\boldsymbol{W'} = \boldsymbol{W}$;
for h *times* **do**
  Let $j \sim \text{UniformInteger}[1, d]$;
  Let $g \sim \text{Uniform}[0, 1]$;
  if $g > 0.05$ **then**
    Simulate $W'_j = W_j * \exp\left[\text{N}(0, \sigma_j)\right]$
    $\delta = \frac{\boldsymbol{W'}}{\boldsymbol{W}}$
  end
  else
    Simulate $W'_j = \text{N}(W_j, 0.1)$
    $\delta = 1$
  end
  Let $\boldsymbol{W'} = \boldsymbol{W'}/|\boldsymbol{W'}|$;
  Accept $\boldsymbol{W} = \boldsymbol{W'}$ with probability $1 \wedge \frac{f(\boldsymbol{W'}|\boldsymbol{a})}{f(\boldsymbol{W}|a)} \cdot \delta$ and update ρ_j ;
  if $h \mod 50 = 0$ **then**
    if $\rho_j > 0.44$ **then**
      $\sigma_j = \exp\left[\log(\sigma_j) + \left(0.05 \wedge \frac{1}{\sqrt(z)}\right)\right]$
    end
    else
      $\sigma_j = \exp\left[\log(\sigma_j) - \left(0.05 \wedge \frac{1}{\sqrt(z)}\right)\right]$
    end
  end
end

Algorithm 2: Constrained adaptive multisite logarithmic random walk used for Dirichlet-distributed random variables.

Data: Human case counts $\boldsymbol{y}^{\star} = \sum_{t,l}\{y_{1tl}, \ldots, y_{ntl}\}$,
source intensities $\boldsymbol{\lambda}^{\star} : \lambda_i^{\star} = \sum_{t,l} \boldsymbol{\alpha}_{tl}^{T}(\boldsymbol{r}_{it} \odot k_t)$
Input: $\mathcal{H}$ the set of cluster identifiers, $\boldsymbol{c}$ an n-dimensional vector of group allocators, $c_i \in \mathcal{H}$, $\boldsymbol{\theta}$ a $|\mathcal{H}|$-dimensional vector of cluster values

```
// Update group allocation c
for i in 1 : n do
    Sample c_i from k(c_i|·) ~ Multinomial(⟨p_h : h ∈ H, p_h*⟩) where
```

$$p_h = |\mathcal{H}_h^{(-i)}| L(y_i^{\star}|\theta_h, \lambda_i^{\star}), \qquad h \in \mathcal{H} \tag{1}$$

$$p_{h^{\star}} = a_q \int_{\Theta} L(y_i^{\star}|\theta, \lambda_i^{\star}) dP_0(\theta), \qquad h \notin \mathcal{H} \tag{2}$$

```
    ;
    if c_i = h* then
        Set H = {H, h*} ;
        Sample θ_h* ~ Gamma(y_i* + a_θ, 1 + b_θ) ;
    end
    else if |H_h| = 0 then
        Set H = H^(-h) ;
    end
end
// Update cluster values θ
for h in H do
    Update θ_h ~ Gamma(∑_{i:c_i=h} y_i* + a_θ, n_h + b_θ)
end
```

Algorithm 3: Marginal Gibbs sampling algorithm using the Chinese Restaurant Process construction of a Dirichlet process

References

[1] Roberts GO, Rosenthal JS. Coupling and Ergodicity of Adaptive Markov Chain Monte Carlo Algorithms. J. Appl. Prob. 2007;44:458–475.
