# Supplementary material for: sourceR: Classification and source attribution of infectious agents among heterogeneous populations
Source: PLoS Comput Biol. 2017 May 30;13(5):e1005564. doi: 10.1371/journal.pcbi.1005564 (PMC5473572; doi:10.1371/journal.pcbi.1005564)
Supplement: S2 Appendix — (PDF) [file pcbi.1005564.s002.pdf]

S2 Appendix. Island model overview

In the Asymmetric Island Model [1, 2], the evolutionary processes (mutation, migration and recombination) of the sequence types are modelled to infer probabilistically the source of each human infection using genetic data from each subtype. The extra information in the genetic typing allows the model to attribute human cases from a type not observed in any sources to a likely source of infection by comparing the genetic similarity to other types that are observed in the sources (this is not possible using the Hald and Modified Hald models). However, this model makes strong assumptions about the evolutionary process (for example, constant molecular clocks) and the uncertainty in these assumptions is not easy to quantify. As the Island model requires sequence data, it cannot be used for source attribution where subtyping is based on phenotypic characteristics, such as serotype.

References

[1] Wilson D, Gabriel E, Leatherbarrow A, Cheesebrough J, Hart C, Diggle P. Tracing the Source of Campylobacteriosis. PLoS Genetics. 2008;.

[2] Wilson D. `iSource`; 2016. Available from: `http://www.danielwilson.me.uk/iSource.html`.
