# Supplementary material for: sourceR: Classification and source attribution of infectious agents among heterogeneous populations
Source: PLoS Comput Biol. 2017 May 30;13(5):e1005564. doi: 10.1371/journal.pcbi.1005564 (PMC5473572; doi:10.1371/journal.pcbi.1005564)
Supplement: S3 Appendix — (PDF) [file pcbi.1005564.s003.pdf]

S3 Appendix. Dutch model overview

The Dutch method [1] is one of the simplest models for source attribution. It compares the number of reported human cases caused by a particular subtype with the relative occurrence of that subtype in each source. The number of reported cases per subtype and reservoir is estimated by:

$$\lambda_{ij} = \frac{r_{ij}}{\sum_j r_{ij}} y_i \tag{1}$$

where r_{ij} is the relative occurrence of subtype i in source j, y_i is the estimated number of human cases of type i per year, λ_{ij} is the expected number of cases per year of type i from source j. A summation across types gives the total number of cases attributed to source j, denoted by ξ_j:

$$\xi_j = \sum_i \lambda_{ij} \tag{2}$$

As the Dutch model has no inherent statistical noise model, confidence intervals for the estimated total attributed cases $\hat{\xi}_j$ by bootstrap sampling over the data set. This model implicitly assumes that there are no source or type specific effects (such as differing virulence of types, or differing consumption of food sources) which is not plausible for most zoonoses.

References

[1] van Pelt W, van de Giessen A, van Leeuwen W, Wannet W, Henken A, Evers E. Oorsprong, Omvang en Kosten van Humane Salmonellose. Deel1. Oorsprong van Humane Salmonellose met Betrekking tot Varken, Rund, Kip, ei en Overige Bronnen. Infectieziekten Bull. 1999;.
