# Supplementary material for: sourceR: Classification and source attribution of infectious agents among heterogeneous populations
Source: PLoS Comput Biol. 2017 May 30;13(5):e1005564. doi: 10.1371/journal.pcbi.1005564 (PMC5473572; doi:10.1371/journal.pcbi.1005564)
Supplement: S4 Appendix — (PDF) [file pcbi.1005564.s004.pdf]

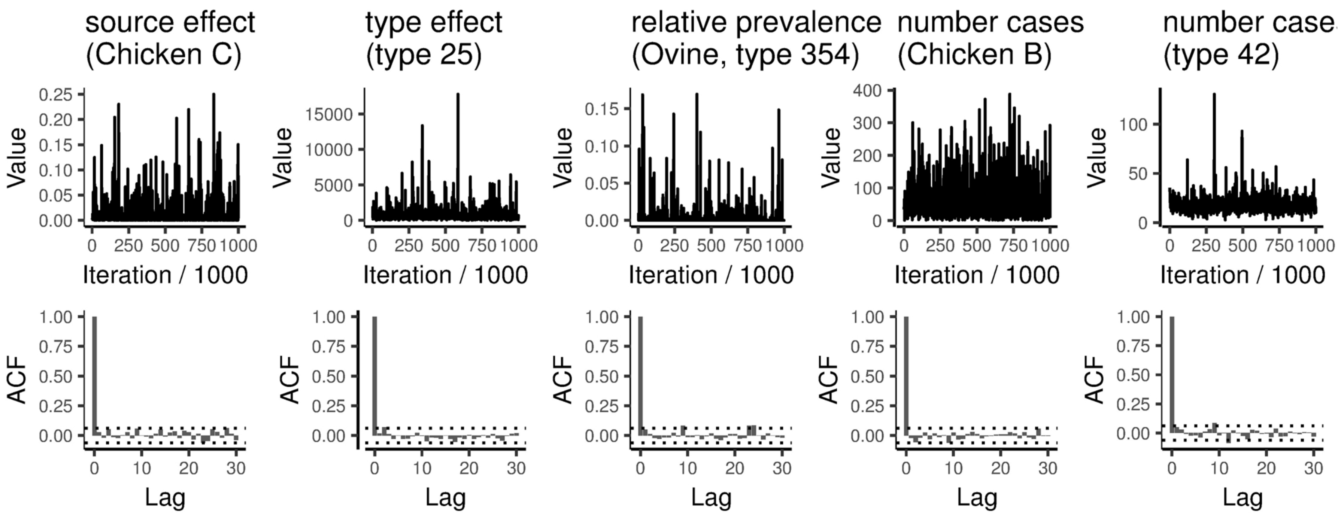

Figure A. Trace and acf plots for a sample of the model parameters.

S4 Appendix. Model fit and convergence diagnostic plots

The trace and autocorrelation plots for a sample of the model parameters show that the model has converged (note, plots were assessed for other parameters to conclude that the model had converged). Comparing the marginal posteriors for each of the λ_i parameters with the associated observed number of human cases shows that the model fits well.

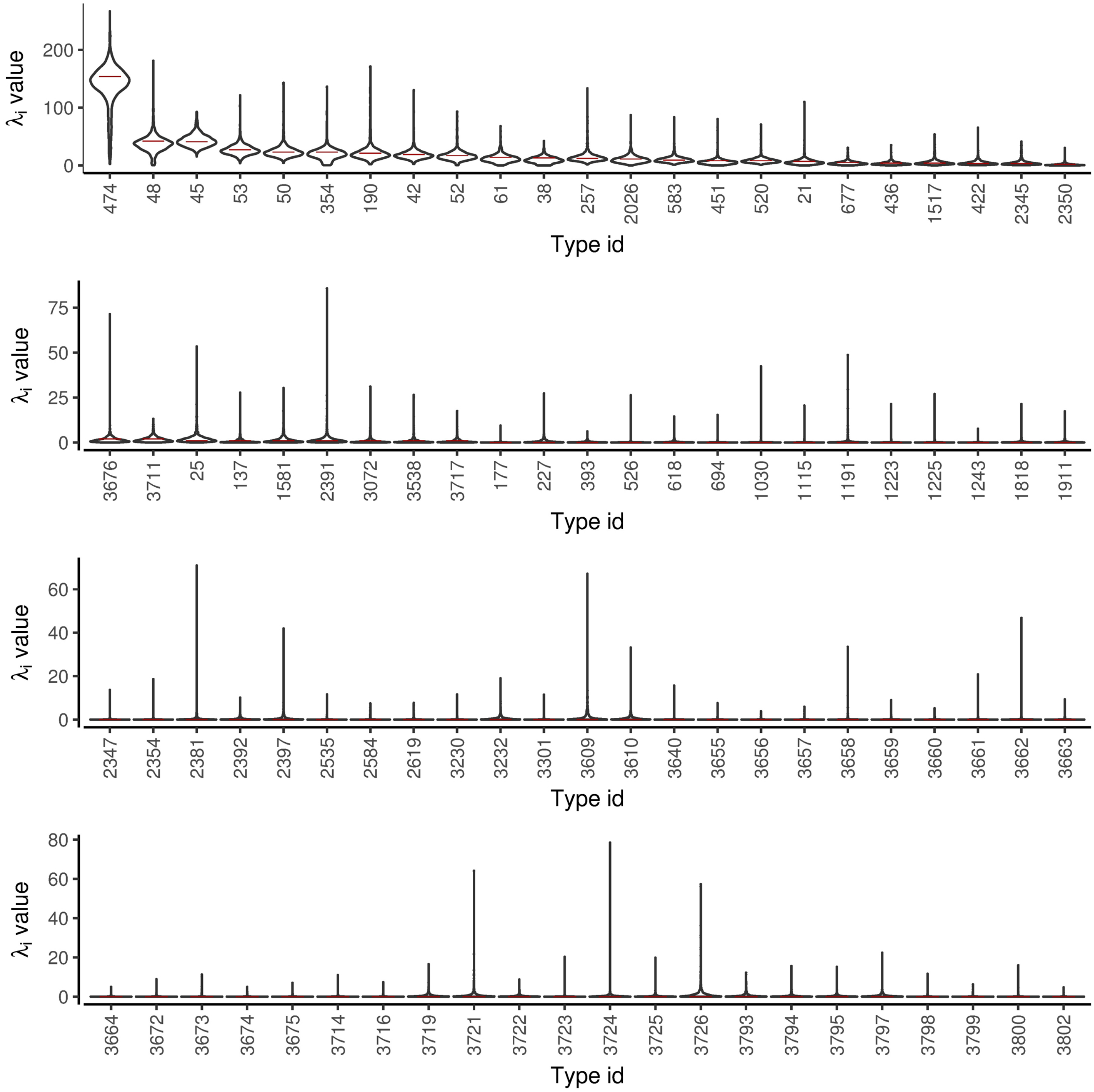

Figure B. Violin plots showing the marginal posterior of each λ_i (estimated number of cases attributed to each type). Observed number of cases for each type are shown as horizontal red lines.
